# Exaggeration of Language-Specific Rhythms in English and French Children's Songs

**DOI:** 10.3389/fpsyg.2016.00939

**Published:** 2016-06-21

**Authors:** Erin E. Hannon, Yohana Lévêque, Karli M. Nave, Sandra E. Trehub

**Affiliations:** ^1^Department of Psychology, University of Nevada Las VegasLas Vegas, NV, USA; ^2^Centre de Recherche en Neurosciences de Lyon, Institut National de la Santé et de la Recherche Médicale, Lyon 1 UniversityLyon, France; ^3^Department of Psychology, University of Toronto MississaugaMississauga, ON, Canada

**Keywords:** rhythm, development, infancy, music, speech, infant-directed modification

## Abstract

The available evidence indicates that the music of a culture reflects the speech rhythm of the prevailing language. The normalized pairwise variability index (nPVI) is a measure of durational contrast between successive events that can be applied to vowels in speech and to notes in music. Music–language parallels may have implications for the acquisition of language and music, but it is unclear whether native-language rhythms are reflected in children's songs. In general, children's songs exhibit greater rhythmic regularity than adults' songs, in line with their caregiving goals and frequent coordination with rhythmic movement. Accordingly, one might expect lower nPVI values (i.e., lower variability) for such songs regardless of culture. In addition to their caregiving goals, children's songs may serve an intuitive didactic function by modeling culturally relevant content and structure for music and language. One might therefore expect pronounced rhythmic parallels between children's songs and language of origin. To evaluate these predictions, we analyzed a corpus of 269 English and French songs from folk and children's music anthologies. As in prior work, nPVI values were significantly higher for English than for French children's songs. For folk songs (i.e., songs not for children), the difference in nPVI for English and French songs was small and in the expected direction but non-significant. We subsequently collected ratings from American and French monolingual and bilingual adults, who rated their familiarity with each song, how much they liked it, and whether or not they thought it was a children's song. Listeners gave higher familiarity and liking ratings to songs from their own culture, and they gave higher familiarity and preference ratings to children's songs than to other songs. Although higher child-directedness ratings were given to children's than to folk songs, French listeners drove this effect, and their ratings were uniquely predicted by nPVI. Together, these findings suggest that language-based rhythmic structures are evident in children's songs, and that listeners expect exaggerated language-based rhythms in children's songs. The implications of these findings for enculturation processes and for the acquisition of music and language are discussed.

## Introduction

Music and language are universal and uniquely human, yet they exhibit tremendous cultural diversity. One consequence of this diversity is that children must acquire culture-specific knowledge and skills without explicit instruction and within a relatively short developmental window. Young listeners must also disentangle musical from linguistic input despite many overlapping elements and features. Because rhythm is prominent in music and language but variable across cultures, it is a potentially important source of information about culture-specific content and structure.

Rhythmic behaviors are ubiquitous in the form of dancing and coordinated music- making. Simple rhythmic patterns and regular underlying beats predominate across cultures (Savage et al., 2015) and are readily perceived by young infants (Trehub and Thorpe, 1989; Baruch and Drake, 1997; Hannon and Johnson, 2005; Winkler et al., 2009; Otte et al., 2013). Nevertheless, there is considerable variation in the complexity and regularity of musical rhythm and beat across cultures (Temperley, 2000; Clayton, 2001). These cross-cultural differences have consequences for music perception and production among adult listeners, even those with no formal music training (Magill and Pressing, 1997; Hannon and Trehub, 2005a; Hannon et al., 2012a; Ullal-Gupta et al., 2014). Importantly, features of culture-specific rhythms gradually influence children's perception of music during a prolonged developmental window (Hannon and Trehub, 2005b; Gerry et al., 2010; Soley and Hannon, 2010; Hannon et al., 2011, 2012b).

Rhythm is also a basic feature of spoken language. The diversity of rhythm and stress patterning across languages of the world gives rise to the perception and production of accent (Cutler, 2012). Listeners use language-specific rhythms to segment words from fluent speech (Vroomen et al., 1998; Ling et al., 2000; Sanders and Neville, 2000), beginning in infancy (Jusczyk et al., 1999; Johnson and Jusczyk, 2001; Thiessen and Saffran, 2003; Thiessen et al., 2005). As in music, rhythm in spoken language is hierarchically structured, with alternating patterns of stressed and unstressed elements occurring at nested hierarchical levels (Liberman and Prince, 1977; Fletcher, 2010). Linguists have grouped languages into rhythmic classes, on the basis that some languages, such as Spanish, give the impression of a machine-gun rhythm, while others, such as English, have a Morse Code quality. Accordingly, syllable-timed languages like Spanish and French were thought to have regular or *isochronous* intervals between syllables, whereas stress-timed languages such as English and Dutch were thought to have isochronous intervals between stressed syllables (and still other mora-timed languages, like Japanese, have the mora as the isochronous unit; Abercrombie, 1967; Cummins, 2009).

Even very young listeners are sensitive to these rhythmic classes. Rhythmic input is available prenatally because of the low-pass filtering properties of the intrauterine environment (Gerhardt and Abrams, 1996; Ullal-Gupta et al., 2013). Prenatal exposure may underlie newborns' preferences for maternal speech (DeCasper and Fifer, 1980; Cooper and Aslin, 1989), their native language (Mehler et al., 1988; Moon et al., 1993), specific passages of speech (DeCasper and Spence, 1986) and specific songs (Hepper, 1991). A role for rhythm in such preferences is implicated by the finding that newborns can discriminate two languages from contrasting rhythmic classes (e.g., Spanish and English) but not from the same rhythmic class (e.g., English and Dutch) even when one is the ambient language (Nazzi et al., 1998). It would seem that language rhythms direct infants' attention to the native language.

The basis for impressions that some languages sound like a machine gun and others like Morse Code is unclear. Acoustic analyses do not support traditional notions of isochronous syllables or stress feet (Dauer, 1983; Grabe and Low, 2002). However, measures of durational contrast between vocalic and consonantal intervals capture some of the presumed differences in language rhythms (Ramus et al., 1999; Grabe and Low, 2002). One such measure is the normalized Pairwise Variability Index (nPVI), which is high in stress-timed languages, where vowel reduction is prominent, and low in syllable-timed languages, where vowel reduction is minimal (Ramus et al., 1999; Grabe and Low, 2002; White and Mattys, 2007).

Speech rhythm tends to govern poetic forms in different languages. For example, English poetic forms such as limericks are organized around the stress foot, whereas French poetic forms are organized around the syllable (Cutler, 2012). One might therefore expect the music of a particular culture to reflect the language rhythms of that culture. Indeed, when the nPVI metric is applied to sequential note durations in instrumental classical and folk music, durational contrast in music parallels speech rhythm from the same region, with, for example, higher nPVIs reported for English than for French music (Huron and Ollen, 2003; Patel and Daniele, 2003; London and Jones, 2011; McGowan and Levitt, 2011). Importantly, non-musician adults accurately classify songs according to their language of origin (French or English), and nPVI predicts how well they generalize this classification to novel songs (Hannon, 2009).

The finding that music and language have parallel rhythmic structure raises important questions about development and learning. Early biases toward familiar stimuli presumably influence what infants learn and when they learn it (Tardif, 1996; Kuhl, 2007; Imai et al., 2008). Given that rhythm may drive infants' early listening biases (Nazzi et al., 1998; Soley and Hannon, 2010), the presence of overlapping rhythms in speech and song input could influence both music and language learning. It could also have implications for their ability to differentiate music from speech, because young listeners would presumably need to use features other than rhythm to accomplish this, such as pitch (Vanden Bosch der Nederlanden et al., 2015). A major goal of the present study was to determine whether the rhythmic differences between stress- and syllable-timed languages have parallels in children's songs.

Child-directed songs from different cultures might preserve or even exaggerate the rhythmic differences between languages. When compared to adult-directed speech, infant- and child-directed speech exaggerate prosodic cues to word boundaries such as stress (Christiansen et al., 1998; Dominey and Dodane, 2004; Thiessen et al., 2005). The typical diminutive forms of the child-directed register in stress-timed languages, such as “mommy” and “doggie,” increase the prevalence of stressed/unstressed syllables, which would predict higher durational contrast in child-directed than in adult-directed speech (Kempe et al., 2005). If exaggerated language-specific rhythms occurred in child-directed linguistic *and* musical input, this would support the hypothesis that music signals cultural or social group membership by reinforcing culturally or linguistically relevant information (Kandhadai et al., 2014; Mehr et al., 2016).

However, child-directed speech is not invariably didactic, and the acoustic features that characterize this style of speaking (and presumably singing) may instead reflect universal caregiving functions such as soothing or emotion regulation (Corbeil et al., 2015; Trehub et al., 2015). Across cultures, infant- and child-directed speech is characterized by higher pitch, greater pitch range, shorter and simpler utterances, slower speech rate, longer pauses, repetition, and rhythmic regularity (Fernald et al., 1989). Although infant-directed singing is more restricted by discrete pitches and rhythmic values, it has many of the features of infant-directed speech, in particular, rhythmic regularity and slower tempos (Trainor et al., 1997; Longhi, 2009; Nakata and Trehub, 2011). As a result, caregiving functions could decrease rhythmic contrasts in child-directed speech and song, regardless of language.

The literature is currently unclear regarding these contrasting predictions. In one study, child-directed speech, regardless of language, had lower nPVIs than adult-directed speech (Payne et al., 2009), whereas other studies found no differences in rhythmic contrast between adult- and child-directed speech for English (Wang et al., 2015) and Japanese (Tajima et al., 2013). In other instances, rhythmic differences between stress-timed and syllable-timed languages were preserved but not exaggerated in child-directed speech and singing (Payne et al., 2009; Salselas and Herrera, 2011). Importantly, prior evidence of higher nPVI in English than in French music was based primarily on instrumental music (Patel and Daniele, 2003). By contrast, one study found no nPVI differences between French and German vocal music (VanHandel and Song, 2010, but see Daniele and Patel, 2013). It is therefore unclear whether children's songs would be expected to exhibit rhythmic features consistent with their language of origin.

The present investigation asked whether children's songs originally set to French or English lyrics exhibit rhythmic patterning in line with their language of origin, as demonstrated for instrumental music. We analyzed rhythmic contrast (as nPVI) in a large corpus of songs from anthologies of children's music. Because children's songs are invariably set to text, we also analyzed a corpus of folk songs also set to text but not designated as children's songs. This allowed us to compare similar genres of vocal music that differed primarily in child-directedness. To determine whether listeners link rhythm to the child-directedness of songs, we collected ratings of instrumental renditions of each song from individuals with different linguistic and cultural backgrounds.

## Corpus analysis

### Materials and methods

The corpus consisted of 269 songs originally set to English or French lyrics. Approximately half were children's songs (English: *n* = 68, French: *n* = 61); the others were folk songs primarily for adults (English *N* = 72, French *N* = 68). Songs were collected from anthologies of folk and children's songs and from Internet sources that provided musical notation and.mid files (See Appendix A for the sources for songs in the corpus). We excluded two songs with the same tune and rhythm as other songs in the corpus but different lyrics). We designated three songs that appeared in folk and children's anthologies as children's songs.

The nPVI provides a measure of durational contrast or rhythmic variability, as shown in the following equation (Grabe and Low, 2002; Patel et al., 2006):

$$\text{nPVI}=\frac{100}{m-1}\times \sum_{k\;=\;1}^{m\;-\;1}\left|\frac{\frac{d_{k}\;-\;d_{k\;+\;1}}{d_{k}\;+\;d_{k\;+\;1}}}{2}\right|$$

where *m* is number of elements in a sequence and *d*_*k*_is the duration of the *k*th element. In the speech literature the *k*th element can be defined by any unit of interest (whether vocalic or consonantal; Ramus et al., 1999; Grabe and Low, 2002). In studies of music, however, the *k*th element is defined exclusively according to musical note duration, or the inter-onset interval between consecutive notes (Patel and Daniele, 2003; Patel et al., 2006). Thus, in line with prior research, the absolute difference was calculated between each successive inter-onset interval, normalized by the mean duration of the pair Values of nPVI range from 0 to 200, with 200 reflecting maximum durational contrast. Note durations were entered directly into a spreadsheet from the sheet music or score, and these duration values were used to calculate nPVI values for each song. Because many songs contained multiple sub-phrases, we omitted values for note pairs that straddled phrase boundaries.

The songs in the corpus were arranged in a variety of meters, including 2/2 (2 English, 0 French), 2/4 (26 English, 55 French), 3/4 (29 English, 23 French), 3/8 (1 English, 3 French), 6/8 (6 English, 20 French), and 4/4 (76 English, 20 French), with 2/4, 3/4, and 4/4 predominating in English and French songs. The corpus included songs composed in all 12 major keys and a few minor keys; C major (40 English, 13 French), F major (30 English, 24 French), and G major (35 English, 49 French) were the most common.

### Results and discussion

On average, nPVI values were higher for English songs (*M* = 42.04, SME = 1.4) than for French songs (*M* = 36.96, SME = 1.5), which is consistent with prior studies of instrumental music by English and French composers (Patel and Daniele, 2003). We also found that nPVI values were lower for children's songs (*M* = 37.12, SME = 1.46) than for folk songs (*M* = 41.88, SME = 1.4), which is consistent with the observation that infant-directed singing has simple, repetitive rhythmic structures and greater temporal regularity than songs performed alone or for adults (Trainor et al., 1997; Longhi, 2009; Nakata and Trehub, 2011).

**Figure 1:**
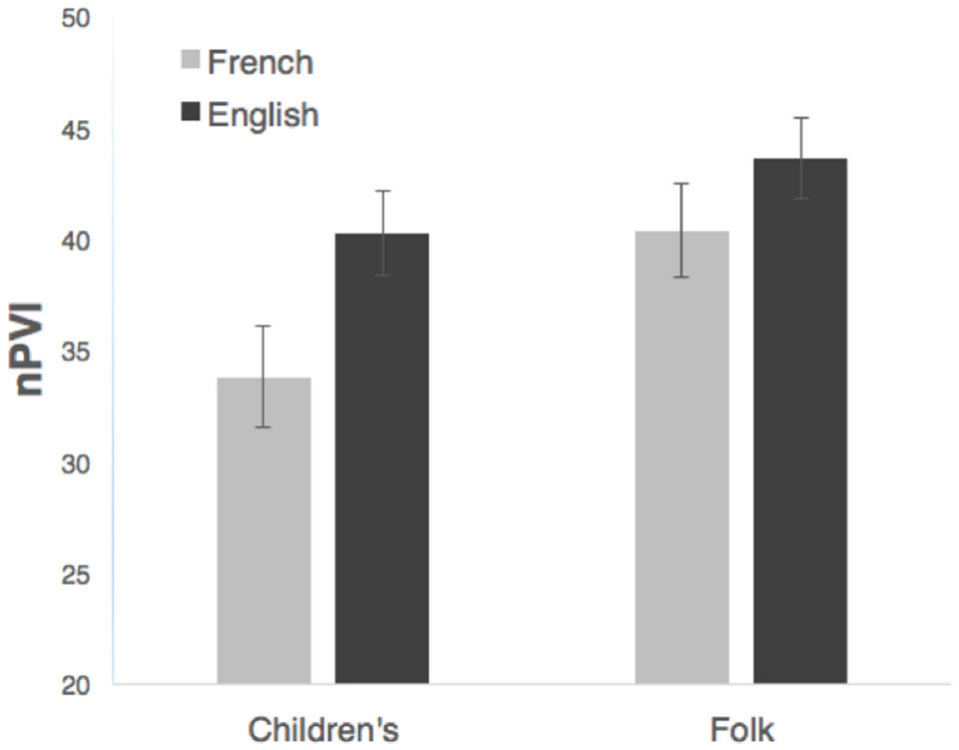
**Durational contrast (nPVI) of songs as a function of language of origin (French or English) and song type (children's or folk)**. Error bars represent standard error.

Although both song types exhibited a trend toward higher English than French nPVI, Figure 1 suggests that this difference was larger for children's songs than for folk songs. Independent samples *t*-tests confirmed that nPVI values were significantly higher for English than for French children's songs (English *M* = 40.43, SME = 1.9; French *M* = 33.8, SME = 2.26), *t*_(127)_=2.256, *p* = 0.026. For folk songs the same trend was evident but not significant, (English *M* = 43.6, SME = 1.8; French *M* = 40.1, SME = 2.13), *t*_(138)_ = 1.27, *p* = 0.10.

This suggests that while English songs in our corpus generally had higher rhythmic variability than French songs, the difference was exaggerated in children's songs. This finding could arise from a tendency to exaggerate native language rhythms in children's songs, at least for syllable-timed languages, either because song creators intuitively exaggerate the rhythms of child-directed lyrics or because of caregivers' intuitive tendency to select children's songs that preserve or exaggerate native-language rhythms. Note that English children's and folk songs did not differ in rhythmic contrast, much like the absence of rhythmic differences between child- and adult-directed English speech (Wang et al., 2015). Native language prosody may constrain child-directed songs such that rhythmic features of the child-directed register, like greater rhythmic regularity and simplicity, are exaggerated in children's songs only when such features preserve native-language prosody. This is consistent with our observation that French children's songs had the lowest nPVI, a trend that was reduced for English.

Our corpus analysis revealed rhythmic differences between children's songs and folk songs, but this interpretation rests on the assumption that songs were classified accurately based on their presence or absence in anthologies of children's music. In prior studies of child-directed speech or singing, the intended audience was clearly known to the speaker or singer (e.g., Fernald, 1985; Nakata and Trehub, 2011), but the situation is less clear for corpora of transcribed songs. Furthermore, some songs occurred in both anthologies of children's and folk songs. At times, caregivers sing pop songs and their own invented songs to infants (Trehub et al., 1997). It is therefore unclear whether native-language rhythm would influence the songs that caregivers choose to perform for children. To further examine this question, we collected ratings of adults from different cultures.

## Ratings

English- and French-speaking adults listened to instrumental versions of the entire corpus from Study 1 and rated each song's familiarity, whether or not they liked it, and whether or not it was “for children.” We expected listeners to be more familiar with and to better like songs from their own culture than from another culture, but the critical question was whether they would be more likely to classify songs as “for children” if they exemplified the rhythms of their native language.

### Materials and methods

#### Participants

All subjects were approved by and run in accordance with the guidelines and principles of the internal review board/ethics committee at University of Nevada Las Vegas and Lyon University. Listeners were recruited from the United States and France. Adults (50 female, 50 male) with self-reported normal hearing from the University of Nevada, Las Vegas participated for partial course credit (*M* = 20.04 years, *SD* = 3.41 years, Range: 18–45 years). The majority of American participants were monolingual native speakers of English s (*n* = 62). The remaining participants were bilingual speakers of English and Spanish (*n* = 24), Italian (*n* = 2), Tagalog (*n* = 5), Polish (*n* = 1), Arabic (*n* = 3), Chamorro (*n* = 1), Korean (*n* = 1), and Amharic (*n* = 1). Bilingual speakers acquired English simultaneously with the other language (*n* = 7) or learned English later as a second language (*N* = 31). Because we were interested in the influences of nationality (country of residence) and native exposure to a syllable-timed language, we created a group of American bilingual participants who acquired a syllable-timed language from infancy (Spanish, Italian, or Tagalog, *n* = 30). The remaining American participants were considered English monolinguals (*N* = 70)^1^. The amount of formal training on a musical instrument ranged from 0 to 16 years (*M* = 2.57 years, *SD* = 3.8 years) for monolingual English speakers and from 0 to 12 years (*M* = 2.26 years, *SD* = 3.26 years) for bilinguals. Dance training ranged from 0 to 14 years (Mean = 1.37 years, *SD* = 3.03 years) for monolinguals and from 0 to 10 years (Mean = 0.91 years, *SD* = 1.94 years) for bilinguals.

Forty adults (26 female, 14 male) recruited in Lyon, France received token compensation for their participation (*M* = 26.40 years, *SD* = 9.63 years, Range: 19–60 years). All French participants had self-reported normal hearing and were native speakers of French, most of whom claimed to have intermediate to high levels of competency in English (*n* = 38). Formal instrumental training ranged from 0 to 17 years (*M* = 4.68 years, *SD* = 5.49 years). Except for one participant, who had 48 years of dance training, formal dance training ranged from 0 to 10 years (*M* = 1.56 years, *SD* = 2.5 years). Dance training did not differ across language groups, *F*_(2, 139)_ = 1.67, *p* = 0.19, but formal music training (in years) did, *F*_(2, 139)_ = 4.08, *p* = 0.02, with the French participants having significantly more formal music training than monolingual, *p* = 0.04, or bilingual Americans, *p* = 0.04.

#### Stimuli

All 269 songs from the corpus analysis were presented to English- and French-speaking participants. To keep the test session to 1 h or less, we randomly divided the corpus into 5 lists of 54 songs each, ensuring that each list contained at least eight of each of the four song types (English or French children's or folk songs). As a result, no participant heard all songs in the corpus, but each song received ratings from at least 28 participants.

Each song was presented as a simple instrumental (flute) melody without words. Using Logic Pro (see http://www.apple.com/logic-pro/), each song was entered directly from the notation into a MIDI sequencer and converted to AIFF format using the *flute* instrument. All songs were transposed to C major. Quarter-note durations were set to 600 ms or 100 beats per minute (bpm) unless the musical notation specified a particular tempo. This resulted in comparable tempos for children's songs (*M* note duration = 484 ms, SME = 18.8) and folk songs (*M* = 488 ms, SME = 18.1), *F*_(1, 265_)=0.027, *p* = 0.87. Overall, however, English songs were slower (*M* = 524 ms, SME = 18.06) than French songs (*M* = 448 ms, SME = 18.8), *F*_(1, 265)_ = 8.39, *p* = 0.004, η^2^_*p*_ = 0.03, however there was no effect or interaction with song type.

#### Procedure

Participants, who were tested individually, were presented with instructions and stimuli over headphones by means of PsyScope software (Cohen et al., 1993). On each trial, after hearing the entire instrumental rendition of a song, participants were asked to rate its familiarity [“How familiar is the song on a scale of 1 (very unfamiliar) to 7 (very familiar)?”], and, if familiar, to provide the song name. Participants were then asked whether it was a children's song (“Do you think this is a children's song?” Yes or No) and to rate the confidence of that judgment on a scale of 1 (very confident this is NOT a children's song) to 7 (very confident this IS a children's song). Finally, participants were asked to rate how much they liked the song [“How much do you like the song on a scale of 1 (dislike very much) to 7 (like very much)?”] Participants responded to these queries for each of 54 songs over the course of 3 blocks of 18 trials.

Following the test session, participants completed a questionnaire (in English or French) about their linguistic/ethnic background, music training, dance training, and hearing status (normal or not). All procedures were reviewed and approved by the local institutional ethics committees in the United States and France, and informed written consent was obtained from all participants.

### Results and discussion

#### Listener effects on mean ratings

In the first analysis we averaged ratings of familiarity, preference, and song type across songs in each category (French children's, English children's, French folk songs, English folk songs) and compared the performance of individuals with contrasting language background and nationality.

##### Familiarity

Each participant's mean familiarity rating was calculated for each song category. Simple correlations showed that years of dance training were unrelated to familiarity ratings for any song category, but there was a modest correlation between music training and familiarity for French children's songs only, *r*_(138)_ = 0.18, *p* = 0.035. Music but not dance training was therefore included as a covariate in subsequent analyses.

**Figure 2:**
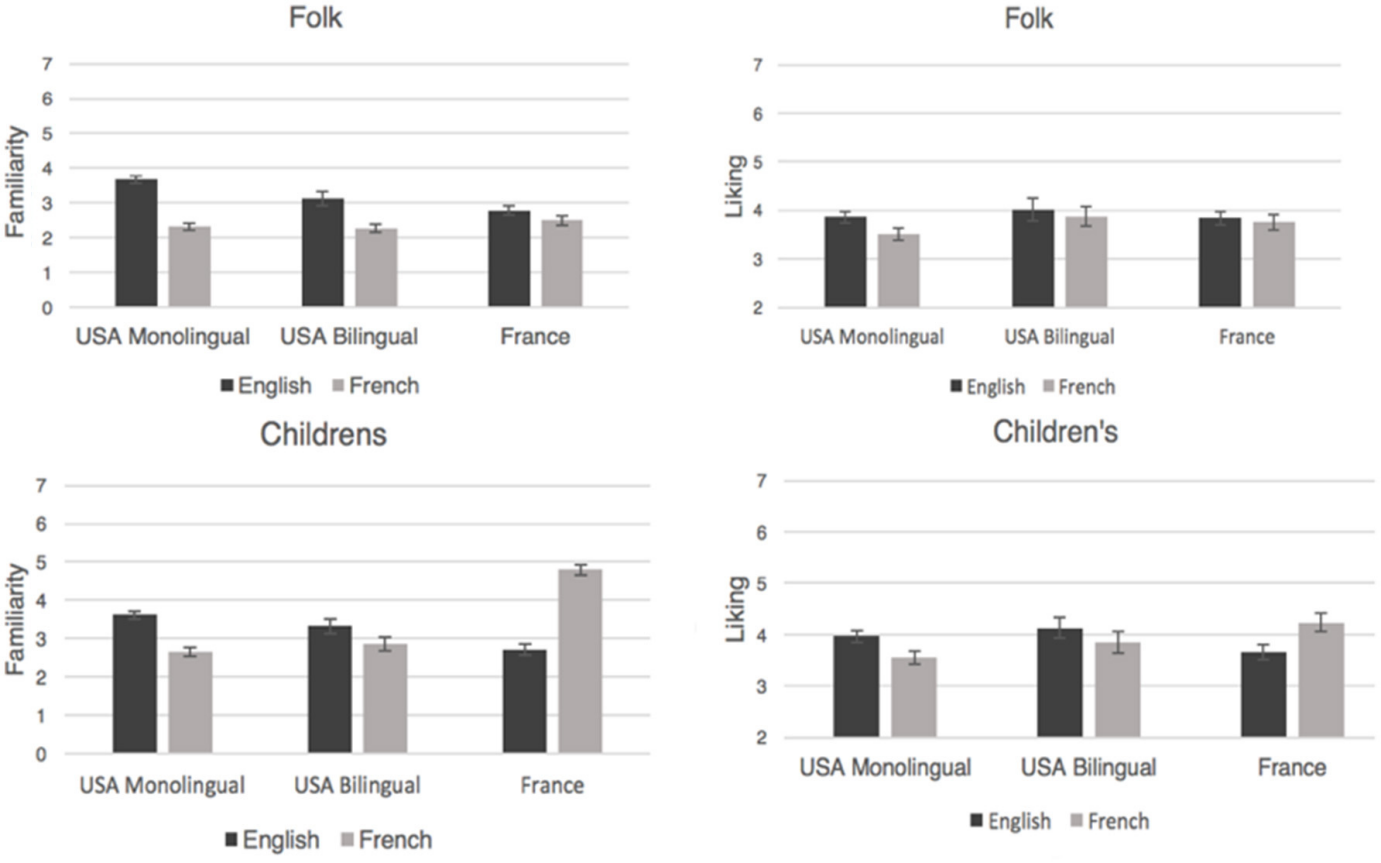
**Mean familiarity ratings (left) and liking ratings (right) of English and French songs by American monolingual, American bilingual, and French listeners, shown separately for folk and children's songs**. Error bars represent standard error.

Overall, songs were considered moderately familiar, with most means falling just below the mid-point of the 7-point rating scale (Figure 2). Familiarity ratings were submitted to a 2 × 2 × 3 (Song Type [children's, folk] × Language of Origin [French, English] × Language Group [monolingual Americans, bilingual Americans, French speakers]) mixed-design analysis of variance (ANOVA), with Music Training (in years) as a covariate. All main effects and interactions for this ANOVA are shown in Table 1. Overall, English songs were significantly more familiar than French songs (English *M* = 3.21, SME = 0.08; French *M* = 2.9, SME = 0.07), and children's songs were rated as significantly more familiar than folk songs (children's: *M* = 3.33, SME = 0.08; folk: *M* = 2.8, SME = 0.07) (Figure 2).

**Table 1:** **Main effects and interactions for all ANOVAs conducted with familiarity rating as dependent variable**.

**Familiarity**	***df***	***F***	**η^2^**p****	***p***
**ALL SONGS**				
Song type^**^	1,136	68.63	0.34	< 0.001
Language of origin^**^	1,136	13.42	0.09	< 0.001
Language group	2,136	1.40	0.02	0.25
Music training	1,136	0.10	0.001	0.75
Song type × language of origin^**^	2,136	70.55	0.34	< 0.001
Song type × language group^**^	2,136	41.00	0.38	< 0.001
Language of origin × language group^**^	2,136	111.8	0.62	< 0.001
Language of origin × music training	2,136	0.41	0.003	0.523
Song type × music training^*^	2,136	5.17	0.04	0.025
Song type × language of origin × language group^**^	2,136	44.3	0.40	< 0.001
Song type × language of origin × music training	2,136	0.36	0.003	0.55
**FOLK SONGS**				
Language of origin^**^	1,136	83.23	0.38	< 0.001
Language group	2,136	2.85	0.04	0.06
Music training	1,136	1.12	0.01	0.29
Language of origin × language group^**^	2,136	25.03	0.27	< 0.001
Language of origin × music training	2,136	0.97	0.007	0.33
**CHILDREN's SONGS**				
Language of origin^*^	1,136	4.28	0.03	0.04
Language group^**^	2,136	7.50	0.10	< 0.001
Music training	1,136	0.16	0.001	0.69
Language of origin × language group^**^	2,136	125.4	0.65	< 0.001
Language of origin × music training	2,136	0.02	0.001	0.90

*Overall ANOVA results are followed by ANOVAs run separately for each Song Type*.

* *p < 0.05*.

** *p < 0.01*.

To ascertain whether listeners were more familiar with songs from their own culture or language, we examined the interactions for Song Type, Language of Origin, and Language Group for each song type (children's or folk) by means of separate 2 × 3 (Language of Origin [English, French] × Language Group [monolingual Americans, bilingual Americans, French]) mixed-design ANOVAs, with Music Training as a covariate.

For folk songs, there was a significant main effect of Language of Origin and a significant interaction between Language of Origin and Language Group (see Table 1). English folk songs were more familiar than French folk songs (English *M* = 3.2, SME = 0.08; French *M* = 2.37, SME = 0.07) for all three groups [monolingual Americans, *t*_(69)_ = 14.2, *p* < 0.001; bilingual Americans, *t*_(29)_ = 6.4, *p* < 0.001; French, *t*_(39)_ = 2.4, *p* = 0.02], but particularly for Americans.

For children's songs there were significant main effects of Language of Origin and Language Group, and an interaction between Language of Origin and Language Group (see Table 1). Higher familiarity ratings were given to French (*M* = 3.44, SME = 0.09) than to English (*M* = 3.22, SME = 0.09) songs. French speakers gave higher overall ratings (*M* = 3.75, SME = 0.14) than monolingual Americans (*M* = 3.14, SME = 0.10) or bilingual Americans (*M* = 3.1, SME = 0.15), *p* < 0.001, and the two American groups did not differ, *p* = 0.81. The interaction in Figure 2 indicates that French listeners were more familiar with French songs than with English songs, *t*_(39)_ = −10.38, *p* < 0.001, and both American groups were more familiar with English songs than with French songs [monolingual Americans, *t*_(69)_ = 9.9, *p* < 0.001; bilingual Americans, *t*_(29)_ = 3.3, *p* = 0.003].

Relatively few participants provided specific names for songs they found familiar, but the percent of correct naming roughly paralleled the observed pattern of familiarity ratings. Monolingual Americans correctly named more English folk (8%) and children's songs (15%) than French folk (<1%) and children's songs (2.5%), as did bilingual Americans (4% English folk, 12% English children's, < 1% French folk, 2% French children's). French listeners, by contrast, correctly named only 1% of English folk songs and 2% of English children's songs, but they correctly named 3% of French folk songs and 28% of French children's songs.

To summarize, familiarity ratings depended primarily on country of residence. Americans found English songs more familiar than French songs, even when their native language was syllable-timed (e.g., bilingual Americans). While all listeners found English folk songs to be more familiar than French folk songs, this difference was smallest for French listeners. In general, familiarity ratings reflected listeners' country of residence, but this pattern was particularly robust for children's songs, with American listeners giving higher ratings to English than French songs and French listeners doing the opposite. This result underscores the prominence of children's songs in everyday listening experience.

##### Liking

Mean liking ratings were uncorrelated with music or dance training. Liking ratings were submitted to a 2 × 2 × 3 (Song Type [children's, folk] × Language of Origin [French, English] × Language Group [English-speaking Americans, bilingual Americans, French]) mixed-design ANOVA. All main effects and interactions are shown in Table 2. Overall, main effects of Language of Origin and Song Type revealed that English songs received significantly higher liking ratings than French songs (English *M* = 3.9, SME = 0.09; French *M* = 3.8, SME = 0.09), and children's songs received significantly higher liking ratings than folk songs (children's: *M* = 3.9, SME = 0.09; folk: *M* = 3.8, SME = 0.09) (Table 2). To examine significant interactions between Language of Origin and Language Group, and between Song Type, Language of Origin, and Language Group (see Table 2), we ran separate 2 × 3 (Language of Origin [English, French] × Language Group [American monolinguals, American bilinguals, French]) mixed-design ANOVAs for each song type.

**Table 2:** **Main effects and interactions for all ANOVAs conducted with liking rating as dependent variable**.

**Liking**	***df***	***F***	**η^2^**p****	***p***
**ALL SONGS**				
Song type^*^	1,137	6.22	0.04	0.014
Language of origin^**^	1,137	7.77	0.05	0.006
Language group	2,137	0.69	0.01	0.50
Song type × language of origin	2,137	3.46	0.03	0.07
Song type × language group	2,137	0.60	0.01	0.55
Language of origin × language group^**^	2,137	24.51	0.03	< 0.001
Song type × language of origin × language group^**^	2,137	10.92	0.014	< 0.001
**FOLK SONGS**				
Language of origin^**^	1,137	15.16	0.10	< 0.001
Language group	2,137	0.68	0.01	0.51
Language of origin × language group^*^	2,137	3.75	0.05	0.026
**CHILDREN's SONGS**				
Language of origin	1,137	0.42	0.003	0.52
Language group	2,137	0.71	0.01	0.50
Language of origin × language group^**^	2,137	26.1	0.28	< 0.001

*Overall ANOVA results are followed by ANOVAs run separately for each Song Type*.

* *p < 0.05*.

** *p < 0.01*.

For folk songs, there was a significant main effect of Language of Origin and a significant interaction between Language of Origin and Language Group (Table 2). English songs were liked more (*M* = 3.9, SME = 0.1) than French songs (*M* = 3.72, *SME* = 0.1). Monolingual Americans gave significantly higher liking ratings to English than to French songs, *t*_(69)_ = 5.09, *p* < 0.001, whereas English and French songs were liked equally by bilingual Americans, *t*_(29)_ = 0.5, *p* = 0.14, and French speakers, *t*_(39)_ = 1.02, *p* = 0.32. Thus, only monolingual Americans preferred folk songs from their own language/culture (Figure 2).

For children's songs, there was a significant interaction between Language of Origin and Language Group. Monolingual Americans gave higher liking ratings to English songs than to French songs, *t*_(69)_ = 5.7, *p* < 0.001, as did bilingual Americans, *t*_(29)_ = 2.8, *p* = 0.009, but French speakers liked French songs more than English songs, *t*_(39)_ = −3.86, *p* < 0.001. Thus, listeners gave higher liking ratings to children's songs whose language of origin matched their country of residence.

Figure 2 suggests that liking ratings paralleled familiarity ratings, which is consistent with evidence that listeners prefer familiar music (Szpunar et al., 2004). However, there were also important differences. Although there were robust effects of nationality on familiarity ratings, there was considerably less variation across groups for liking ratings. This was particularly notable for bilingual Americans and French speakers who rated English folk songs as more familiar than French folk songs but nevertheless did not necessarily like English folk songs better than French folk songs. Thus liking ratings might only partially reflect familiarity.

##### Classification

A measure of perceived “child-directedness” was derived by calculating for each participant the proportion of songs labeled “for children” in each of the four song categories. These values were uncorrelated with dance training, but they were positively correlated with music training for French children's songs only, *r*_(140)_ = 0.18, *p* = 0.03. Therefore music training was included as a covariate in subsequent analyses.

**Figure 3:**
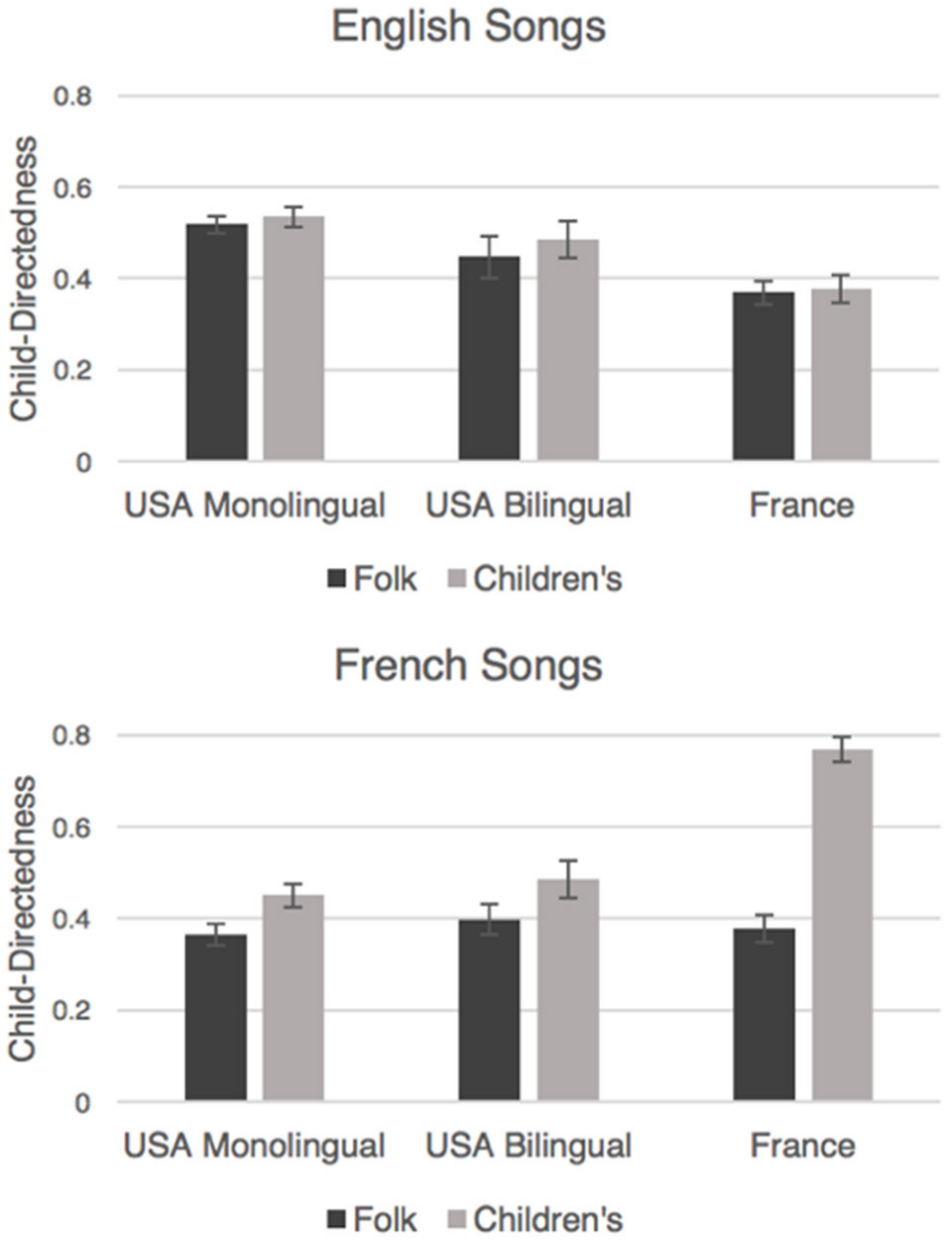
**Proportion of songs in each category labeled as “for children” by American monolingual, American bilingual, and French listeners, shown separately for English (top) and French (bottom) songs**. Error bars represent standard error.

The derived child-directedness measure was submitted to a 2 × 2 × 3 (Song Type [children's, folk] × Language of Origin [French, English] × Language Group [monolingual Americans, bilingual Americans, French]) mixed-design ANOVA, with years of Music Training as a covariate. All main effects and interactions are shown in Table 3. We observed a main effect of Song Type, with overall higher proportions of “for children” classifications given to children's songs (*M* = 0.514, SME = 0.02) than to folk songs (*M* = 0.42, SME = 0.016; see Table 3). There were also significant two-way interactions between Song Type and Language of Origin, Song Type and Language Group, Language of Origins and Language Group, and among Song Type, Language of Origin, and Language Group (see Table 3). Our goal with the child-directedness measure was to examine whether listeners' classifications corresponded to the traditional classifications of children's songs vs. folk songs. For this analysis, we therefore ran separate 2 × 3 (Song Type [children's, folk] × Language Group [English-speaking Americans, bilingual Americans, French]) mixed-design ANOVAs, with Music Training as a covariate. Figure 3 displays adults' ratings of children's and folk songs for each group, separately for each language of origin.

**Table 3:** **Main effects and interactions for all ANOVAs conducted with derived child-directedness rating as dependent variable**.

**Child-directedness**	***df***	***F***	**η^2^**p****	***p***
**ALL SONGS**				
Song type^**^	1,136	32.18	0.19	< 0.001
Language of origin	1,136	1.34	0.01	0.25
Language group	2,136	0.096	0.001	0.91
Music training	1,136	1.05	0.008	0.31
Song type × language of origin^**^	2,136	38.6	0.22	< 0.001
Song type × language group^**^	2,136	11.76	0.15	< 0.001
Language of origin × language group^**^	2,136	54.05	0.44	< 0.001
Language of origin × music training	2,136	0.001	0.00	0.98
Song type × music training	2,136	0.91	0.007	0.16
Song type × language of origin × language group^**^	2,136	21.80	0.24	< 0.001
Song type × language of origin × music training	2,136	0.94	0.007	0.33
**ENGLISH SONGS**				
Song type	1,136	0.02	0.000	0.89
Language group^**^	2,136	11.56	0.15	< 0.001
Music training	1,136	0.91	0.007	0.34
Song type × language group	2,136	0.40	0.006	0.67
Song type × music training	2,136	2.04	0.015	0.16
**FRENCH SONGS**				
Song type^**^	1,136	63.7	0.32	< 0.001
Language group^**^	2,136	10.17	0.13	< 0.001
Music training	1,136	0.77	0.006	0.38
Song type × language group^**^	2,136	29.1	0.30	< 0.001
Song type × music training	2,136	0.01	0.00	0.95

*Overall ANOVA results are followed by ANOVAs run separately for each Language of Origin*.

* p < 0.05.

** *p < 0.01*.

For English songs, we observed a significant main effect of Language Group (Table 3), with French listeners giving lower child-directedness ratings (*M* = 0.37, SME = 0.03) than monolingual Americans (*M* = 0.53, SME = 0.02), *p* < 0.001, or bilingual Americans (*M* = 0.47, SME = 0.03), *p* = 0.014, who did not differ from each other, *p* = 0.09. There was no indication, however, that any of the groups classified English children's songs as more child-directed than English folk songs.

For French songs, we observed significant main effects of Song Type and Language Group (Table 3). French children's songs received higher child-directedness ratings (*M* = 0.57, SME = 0.02) than French folk songs (*M* = 0.57, SME = 0.02). Moreover, higher child-directedness ratings were given by French speakers (*M* = 0.57, SME = 0.03) than by monolingual Americans (*M* = 0.41, SME = 0.02), *p* < 0.001, or bilingual Americans (*M* = 0.44, SME = 0.03), *p* = 0.004, who did not differ from each other, *p* = 0.39. We also observed a two-way interaction between Song Type and Language Group (Table 3), with Bonferroni-corrected *post-hoc t*-tests revealing that French listeners gave far higher ratings to French children's songs than did monolingual Americans, *t*_(108)_ = −7.9, *p* < 0.001, or bilingual Americans, *t*_(68)_ = −5.8, *p* < 0.001, and the latter two groups did not differ, *t*_(98)_ = −0.77, *p* = 0.44. Despite these differences, all three groups accurately rated French children's songs as more child-directed than French folk songs [monolingual Americans, *t*_(69)_ = 3.6, *p* < 0.001; bilingual Americans, *t*_(29)_ = 2.4, *p* = 0.02; French, *t*_(39)_ = 10.1, *p* < 0.001].

To summarize, listeners' likelihood of endorsing a song as “for children” was higher for children's songs than for folk songs, which validates the traditional classification of songs in the corpus. However, even though this trend was evident for English songs (Figure 3), the main effect of Song Type was driven by French songs, and French listeners showed the most robust differentiation of children's songs from folk songs. This finding is surprising because American listeners, regardless of language background, did not differentiate English children's songs from folk songs, despite greater familiarity with English songs. Instead, American listeners generally rated all English songs as more child-directed than French songs (Figure 3). Perhaps this is not surprising in light of the observation (Figure 1) that French folk and children's songs are better differentiated than English folk and children's songs.

#### Regression analysis

The aforementioned results indicate that, on the whole, songs from children's anthologies were more likely to be classified as “for children,” and listeners were more likely to like songs from their own culture. It is also clear, however, that children's songs were more familiar than folk songs, and songs from one's culture were more familiar than other songs. We therefore conducted multiple regression analyses, one for each language group, to determine the relative contributions of familiarity, preference, and rhythmic features (nPVI and tempo) in predicting the perceived child-directedness of each song.

For each listener group, four variables indicative of rhythmic variability (nPVI), tempo (mean duration of each note in ms), familiarity (mean familiarity rating), and preference (mean liking ratings) were regressed onto the averaged response for each group (tendency to classify a song as “for children”) for each of the 269 songs. Table 4 presents simple correlations for each variable, separately for each group. Multiple regressions were conducted to determine how the removal of specific variables affected the predictiveness of the model. Thus, *R*^2^ Change for a given variable indicates the amount by which the predictive strength of the model containing all four variables decreases when that variable is removed from the regression, reflecting the unique contribution of that variable (Darlington, 1990).

**Table 4:** **Simple (***r***) and ***R***^**2**^ Change predicting child-directedness endorsements from variables rhythm (nPVI), mean note duration (Tempo), Preference, and Familiarity, separately for each listener group**.

	**Predictor variable**	***r***	***R*^2^ change**
	**MONOLINGUAL AMERICAN**			
	Familiarity	0.74^**^	0.20^**^
	Preference	0.59^**^	0
	Tempo	−0.32^**^	0.03^**^
	nPVI	−0.07	0.004
**BILINGUAL AMERICAN**			
	Familiarity	0.57^**^	0.18^**^
	Preference	0.36^**^	0.00
	Tempo	−0.30^**^	0.03^**^
	nPVI	−0.12^*^	0.01^a^
**FRENCH**			
	Familiarity	0.61^**^	0.29^**^
	Preference	0.25^**^	0.004
	Tempo	−0.21^**^	0.014^*^
	nPVI	−0.20^**^	0.022^*^

* *p < 0.05*.

** *p < 0.01*.

a *p = 0.053*.

The four-variable models yielded moderate prediction levels for all three groups [monolingual Americans, *R*${}_{\left(4,\;264\right)}^{2}=0.59$, *p* < 0.001; bilingual Americans, *R*${}_{\left(4,\;264\right)}^{2}=0.38$, *p* < 0.001; French, *R*${}_{\left(4,\;264\right)}^{2}=0.41$, *p* < 0.001]. As shown in Table 4, familiarity and liking were positively correlated with child-directedness, suggesting that listeners had a strong tendency to classify songs that were more familiar and that they liked as “for children.” Of the two measures, only familiarity contributed uniquely and robustly to the models for all three groups (Table 4). This suggests that while liking ratings correlated with child-directedness ratings, liking did not predict child-directedness after controlling for familiarity. Tempo correlated negatively with responses and contributed uniquely to the model for all three groups. Faster songs were rated as more child-directed, a tendency that was somewhat stronger for American than for French listeners. Critically, nPVI did not correlate with the child-directedness ratings of monolingual Americans, but it correlated with the responses of bilingual American and French listeners, such that for these groups lower nPVI was associated with children's songs. Even after controlling for familiarity, nPVI contributed uniquely to the model for French listeners and marginally for American bilingual listeners (Table 4). This suggests that for speakers of a syllable-timed language, rhythmic features predict the perceived appropriateness of a song for children.

## General discussion

The present study is the first to demonstrate that the observed rhythmic parallels between the music and language of different cultures are not only preserved in music for children (children's songs) but also exaggerated relative to a similar genre of music (folk songs). By complementing our corpus analysis with listener ratings, we show that rhythmic differences in our corpus may reflect culture-specific intuitions about the role of rhythm in children's music. Our findings suggest that, when considering a repertoire of songs to perform for or with children, French-, and to some extent, Spanish-speaking listeners are more likely to select a song with lower rhythmic contrast, which parallels and enhances the rhythmic features of their language. By contrast, English-speaking listeners generally endorse English songs regardless of rhythm, which is consistent with the properties of English children's songs and folk songs but results in song choices that maintain the higher rhythmic contrast typical of English.

If children's music reinforces linguistically or culturally relevant information by exaggerating language-specific speech rhythm (Kandhadai et al., 2014), one might expect English children's songs to exhibit greater exaggeration (higher contrast) than English “adult-directed” folk songs, and French children's songs to exhibit greater exaggeration (lower contrast) than French folk songs. Our results are consistent with this prediction for French songs but not for English songs, which had comparable rhythmic contrast for both song types. In child-directedness ratings, moreover, French listeners robustly differentiated French children's songs from folk songs, while English speakers did not do so for English songs. Instead, English speakers, like French and Spanish speakers, instead classified French children's songs as child-directed even though those songs had lower rhythmic contrast (unlike English). The regression analysis indicated, however, that these decisions were driven by song familiarity and tempo rather than rhythm. In other words, English listeners did not use rhythm in their ratings of child-directedness, perhaps because of their exposure to music that is rhythmically undifferentiated across child and folk song categories.

Why is exaggeration of language-typical rhythmic patterns absent in English songs but present in French songs? This situation could represent a trade-off between the caregiving and didactic functions of child-directed input, with increased rhythmic regularity in child-directed vocalizations being universal (Fernald et al., 1989; Trainor et al., 1997) and native- language speech rhythm varying by culture (Payne et al., 2009; Wang et al., 2015). For French input, lower regularity is consistent with both caregiving and didactic functions, whereas for English these two functions are at odds. Perhaps English children's songs would be more rhythmically regular if reduced rhythmic contrast did not undermine native language rhythm.

Cultural differences in caregiving styles may provide yet another explanation for why English children's songs are not more rhythmically regular. North American caregivers engage in more stimulating and playful interaction with infants than do caregivers from other cultures, who are more likely to soothe infants and lull them to sleep (Trehub and Trainor, 1998). While rhythmic contrast in infant-directed speech varies by communicative intention (e.g., affection, disapproval or questions; Salselas and Herrera, 2011), a systematic analysis of rhythmic contrast in play songs and lullabies would shed light on this issue.

The bilingual, English-speaking Americans were expected to disentangle the influence of native language from country of residence because all of them acquired a syllable-timed language from birth yet lived in the United States and presumably had continuous exposure to American music. Indeed, familiarity ratings suggest that this group was very similar to monolingual Americans in their exposure to folk and children's songs. By contrast, their preference ratings were only partially consistent with monolingual Americans, and the regression analysis suggested that bilingual Americans were more likely to use nPVI when endorsing child-directedness in songs, although this result did not reach conventional levels of significance. Further research is needed to ascertain whether exposure to multiple languages and cultures influences the perception and use of rhythm in linguistic and musical interactions with children.

The present work has several limitations. Practical considerations resulted in unequal sample sizes across groups, potentially affecting some outcomes by reducing power, notably the small group of bilingual Americans. Similarly, although our corpus size was comparable to or larger than that in several related studies of music (McGowan and Levitt, 2011; Salselas and Herrera, 2011; Temperley and Temperley, 2011), it was much smaller than that in other studies of music (Huron and Ollen, 2003; Patel and Daniele, 2003) and speech (Payne et al., 2009; Wang et al., 2015). The present study was also limited to French and English materials and to the coarse nPVI measure that may not capture the nuanced rhythmic features that differentiate many other languages (Cutler, 2012). Because of our exclusive reliance on musical notation, our nPVI values may differ in important ways from expressively sung performances, which might further enhance speech rhythms in music (Palmer, 1997). Furthermore, in view of the fact that multiple variability measures have been used in the speech literature (based on vocalic or consonantal durations, for example), it may be worthwhile to consider other units of musical time, for example, using both note duration and inter-onset interval, particularly in performed music. A future goal is to expand the corpora of child-directed music to expressively performed songs in a wider range of cultures.

The rating study was also limited by training differences across groups. French participants had more music training than American participants, and training was positively correlated with some measures (familiarity and child-directedness ratings of French songs). Music training was included as a covariate whenever it was correlated with any measures, and there were no interactions with music training. Nevertheless, musicians may be more sensitive to rhythmic features that distinguish folk songs from children's songs, leading French listeners to outperform American listeners regardless of native language and country of residence. The performance of bilingual Americans casts doubt on this explanation because bilingual Americans' child-directedness endorsements were driven by nPVI, like those of French listeners, despite having less music training. Although it is desirable to balance music training across groups, imbalances in music training are often central to the cultures under consideration.

Overall, the present findings provide new insights into the role of rhythm in music development by indicating that rhythmic features of the native language not only appear in children's music from that culture but are enhanced in such music. Because rhythm is accessible from birth (Winkler et al., 2009) and drives early listening preferences (Nazzi et al., 1998; Soley and Hannon, 2010), the presence of native-language rhythm in musical input may have important implications for learning in music and language domains. In one example of generalization from music to speech processing, 9-month-old infants who participated in a 4-week intervention involving movement to music with triple meter exhibited enhanced neural processing of temporal structure in speech and music relative to infants who participated in a play intervention without music (Zhao and Kuhl, 2016). Incidental exposure to language input in verse or song may fine tune temporal attention and enhance memory, providing a particularly effective scaffold for young children's learning (Levedeva and Kuhl, 2010; de Diego-Balaguer et al., 2016; Kiraly et al., 2016). In sum, rhythmic input affects enculturation and cultural transmission by ensuring that young children are exposed to the communication features of their social and cultural group.

## Author contributions

EH conceived and carried out corpus analysis, EH and ST designed behavioral study, EH supervised and trained research assistants who created stimuli, experiment program, and ran the experiment in the USA, YL collected and supervised research in France, EH, YL, and KN analyzed data, EH, YL, KN, and ST wrote the paper.

### Conflict of interest statement

The authors declare that the research was conducted in the absence of any commercial or financial relationships that could be construed as a potential conflict of interest.

## Footnotes

^1^Because we were interested in the relationship between native-language rhythm and song ratings, we chose to combine monolingual English speakers with the eight bilingual participants who spoke stress-timed languages. Although these eight participants were bilingual, they were like monolingual English speakers in that they acquired a syllable-timed language from birth. All reported analyses without these 8 participants yielded the same results.

## Appendix A. corpus song sources.

**Table:**

**English Song Sources**
Davison, A. T., and Surette, T. W. (1922b). *140 Folk-Tunes: Rote Songs: Grades I, II and III for School and Home*. Boston, MA: E.C. Schirmer Music Co.
Leonard, H. (1998). *The Really Big Book of Children's Songs*. Milwaukee, WL: H. Leonard Corp.
Leonard, H. (1999). *The Mighty Big Book of Children's Songs*. Milwaukee, WL: H. Leonard Corp.
Radcliffe-Whitehead, J. B. (1903). *Folk-Songs and other Songs for Children*. Boston, MA: Oliver Ditson Company.
Seeger, R. C. (1948). *American Folk Songs for Children*. New York, NY: Doubleday & Co.
Haywood, C. (1966). *Folk Songs of the World, Gathered from more than 100 Countries*. New York, NY: J. Day Co.
Lomax, J. A., Lomax, A., and Kittredge, G. L. (1994). *American Ballads and Folk Songs*. New York, NY: Dover.
Lomax, J. A., Lomax, A., Seeger, R. C., Thompson, H. W., and Tick, J. (2000). *Our Singing Country: Folk Songs and Ballads*. Mineola, NY: Dover.
Moore, E., and Moore, C. O. (1964). *Ballads and Folk Songs of the Southwest: More Than 600 Titles, Melodies, and Texts Collected in Oklahoma*. Norman, OK: University of Oklahoma Press.
Trehune, P. (2006). *Guitar Masters Quality Publications* [sheet music in.PDF file format]. *Guitar-Masters*. Available online at: http://guitar-primer.com/Folk/ (Accessed November 28, 2006).
**French Song Sources**
Davison, A. T., and Surette, T. W. (1922a). *140 Folk Tunes for School and Home*. Boston, MA: E.C. Schirmer Music Co.
Momes.net (*trans*. Kids.net). *Comptines (trans:Rhymes)* [sheet music in.PDF file type]. Available online at: http://www.momes.net/comptines/ (Accessed October 20, 2006).
Bujeaud, J. (1980). *Chants et Chansons Populaires des Provinces de l'ouest*. (Trans. Popular chants and songs from the provinces of L'ouest). Marseille: Laffitte Reprints.
Byrd, J. (1903). *Folk Songs and Other Songs for Children*. Boston, MA: Radcliffe-Whitehead.
Fassio, A. (1932). *French Folk Songs: Nursery Rhymes and Children Rounds*. New York, NY: Marks Music Corporation.
Gerhard, R. (2002). *Six Chansons Populaires Francaises: Arrangement Pour Chant et Piano*. (Trans. Six French Folksongs: arranged for voice and piano). London: Boosey & Hawkes.
Karpeles, M., (Ed.) (1956). *Folk Songs of Europe*. London: Novello and Co. Limited.
Loffet, B. (2006). *Plein de tablatures gratuites pour accordéon diatonique* (Trans. Full free tablatures for diatonic accordion) [Sheet music in.PDF file type]. available online at: http://diato.org/tablat.htm (Accessed December 5, 2006)
Macaulay, W. (2004). Five *Line Skink version 1.2a4* [ABC reader program for Mac OSX].
Piron, S. (2007). *655 airs trad. de France (Trans. 655 Traditional Tunes from France) [sheet music in ABC notation]*. Available online at: http://www.tradfrance.com/matf01.txt (accessed January 27, 2007).
Poire, H. (1962). *Mon Premier Livre de Chansons* (Trans. My first song book). Paris: Larousse.
